# Substance use and its predictors among undergraduate medical students of Addis Ababa University in Ethiopia

**DOI:** 10.1186/1471-2458-11-660

**Published:** 2011-08-22

**Authors:** Wakgari Deressa, Aklilu Azazh

**Affiliations:** 1 Department of Epidemiology and Biostatistics, School of Public Health, College of Health Sciences, Addis Ababa University, Ethiopia; 2 Department of Internal Medicine, School of Medicine, College of Health Sciences, Addis Ababa University, Ethiopia

## Abstract

**Background:**

Substance use remains high among Ethiopian youth and young adolescents particularly in high schools and colleges. The use of alcohol, *khat *and tobacco by college and university students can be harmful; leading to decreased academic performance, increased risk of contracting HIV and other sexually transmitted diseases. However, the magnitude of substance use and the factors associated with it has not been investigated among medical students in the country. This study was conducted to determine the prevalence of substance use and identify factors that influenced the behavior among undergraduate medical students of Addis Ababa University in Ethiopia.

**Methods:**

A cross-sectional study using a pre-tested structured self-administered quantitative questionnaire was conducted in June 2009 among 622 medical students (Year I to Internship program) at the School of Medicine. The data were entered into Epi Info version 6.04d and analyzed using SPSS version 15 software program. Descriptive statistics were used for data summarization and presentation. Differences in proportions were compared for significance using Chi Square test, with significance level set at p < 0.05. Multivariate logistic regression analyses were used to assess the magnitude of associations between substance use and socio-demographic and behavioral correlates.

**Results:**

In the last 12 months, alcohol was consumed by 22% (25% males vs. 14% females, p = 0.002) and *khat *use was reported by 7% (9% males vs. 1.5% females, p < 0.001) of the students. About 9% of the respondents (10.6% males vs. 4.6% females, p = 0.014) reported ever use of cigarette smoking, and 1.8% were found to be current smokers. Using multiple logistic regression models, being male was strongly associated with alcohol use in the last 12 months (adjusted OR = 2.14, 95% CI = 1.22-3.76). Students whose friends currently consume alcohol were more likely to consume alcohol (adjusted OR = 2.47, 95% CI = 1.50-4.08) and whose friends' use tobacco more likely to smoke (adjusted OR = 3.89, 95% CI = 1.83-8.30). *Khat *use within the past 12 months was strongly and positively associated with alcohol consumption (adjusted OR = 15.11, 95% CI = 4.24-53.91). Similarly, ever use of cigarette was also significantly associated with alcohol consumption (adjusted OR = 8.65, 95% CI = 3.48-21.50).

**Conclusions:**

Concordant use of alcohol, *khat *and tobacco is observed and exposure to friends' use of substances is often implicated. Alcohol consumption or *khat *use has been significantly associated with tobacco use. While the findings of this study suggest that substance use among the medical students was not alarming, but its trend increased among students from Year I to Internship program. The university must be vigilant in monitoring and educating the students about the consequences of substance use.

## Background

Use of substances such as alcohol, *khat *leaves (*Catha edulis*) and tobacco has become one of the rising major public health and socio-economic problems worldwide. Recent trends indicate that the use of substances have dramatically increased particularly in developing countries [1]. Alcohol, especially in high doses, or when combined with *khat *or tobacco, continues to claim the lives of many people. It is estimated that 9% of the global population aged 12 or older are classified with dependence on psychoactive substances such as alcohol [2]. Heavy consumption of *khat *is associated with euphoria, hyperactivity, anorexia, insomnia, lethargy and depression. In addition, the combined use of alcohol and *khat *could increase sexual risky behavior contributing to the spread of human immuno-deficiency virus (HIV) infection [2].

The problem of substance use has historically been linked to health professionals due to their close proximity to the drugs. This problem highly impairs the practice of medicine with reasonable skill and safety to patients because of their illness or dependence on drugs [3-5]. Tobacco consumption has been the main risk factor for chronic diseases such as cancers, chronic lung disease, diabetes and cardiovascular diseases, however; its use has become a growing concern among college students in many parts of the world [6]. Furthermore, the use of drugs and alcohol among medical students has become a growing concern [7,8]. Studies also suggest differentials in alcohol and other drug use between adolescents and young adults with regard to sexual identity among undergraduate students [9].

With 74 million people in 2007, Ethiopia is the second most populous nation after Nigeria in sub-Saharan Africa with an annual population growth rate estimated at 2.7% [10]. Children under the age of 15 years comprise about 45% of the total population. Several studies indicate that substance use among Ethiopian adolescents is considerably rising [11-14]. Nowadays, alcohol and *khat *are widely consumed among high school and college students in Ethiopia [11,13-15]. There is a strong link between *khat *chewing and excessive alcohol consumption, and it is believed to be one of the factors associated with unprotected risky sexual behavior, predisposing the youth for HIV infection and transmission [14,16,17]. Studies also indicate that adolescent girls start and regularly use drugs and alcohol than boys [18].

Of the young segment of the Ethiopian population, college and university students are the most at risk of using alcohol and other drugs such as *khat *and tobacco. Most often stimulant medications are increasingly used by high school and college students as a means to improve academic performance. Entering the university, often leads to new opportunities, independence from family control, self-decision making, and peer-pressures to use or abuse alcohol or other drugs. It is generally acknowledged that several factors are involved in the initiation of substance use among adolescents and young adults.

The use of alcohol, *khat *and tobacco among adolescents can be harmful, leading to decreased academic performance, increased risk of contracting HIV and other sexually transmitted diseases, or other psychiatric disorders such as lethargy, hopelessness and insomnia [2]. Furthermore, it exposes students to legal repercussions, or jeopardizes their enrollment at the university. Substance use behaviors among medical students have important implications for the health of the general population since physicians and future physicians are important role models in terms of health related behaviors. This group of population should be identified as a crucial group for preventing substance use and the transmission of HIV [19-21].

Though alcohol consumption, *khat *chewing and cigarette smoking have become common practices among high school and college/university students in Ethiopia, only very few studies have assessed their magnitude and the associated factors [11,13]. The practice of medicine requires commitment, enthusiasm and altruism, indicating that medical students deserve special attention in relation to alcohol and other drug use. They are the ones who will be responsible for the health of the society in the future. Therefore, it is important to understand the pattern of substance use among this population group.

The objective of this study was to explore the magnitude of self-reported substance use and any association between these habits and the associated factors among undergraduate medical students. Such information provides crucial support for efforts aimed at developing a healthy lifestyle, strengthening on campus standards of conduct and for improving HIV/AIDS prevention and control programs among college and university students.

## Methods

This study was carried out in June 2009 among undergraduate medical students attending from Year I to internship program at the Faculty of Medicine, Addis Ababa University, Ethiopia. The Faculty of Medicine with its specialized Tikur Anbessa teaching hospital was founded in 1964 in Addis Ababa, the capital of Ethiopia. By the time of the survey, there were 19 departments at the Faculty. In June 2009, the total number of full-time registered undergraduate medical students from Year I to internship was 800; of whom about 36% were females. Since its establishment, the Faculty has graduated 1890 general medical practitioners (MD) and 862 specialists [22]. The duration for the undergraduate medical training has a curriculum lasting for 5 1/2 years and the postgraduate medical training lasts between 2-4 years depending on the area of specialty. The medical school admits students from all over the country and uptake into the school for undergraduate program is determined by the national criteria set by the Federal Ministry of Education based on the performance of high school leaving examinations.

The study utilized an institution-based cross-sectional study design with quantitative data collection method. The study population included all medical students (Year I to internship program) attending medical training at the Faculty of Medicine. There were no pre-medical students registered at the Faculty at the time of the study. The sample size was determined by Epi Info 2002 software package using a single proportion formula for cross-sectional survey, based on the prevalence of alcohol consumption (44%) during the past year among university students [23]. Using 4% margin of error at 95% confidence level, the sample size required was 615 after considering 5% non-response rate. Since the number of all medical students across all batches at the Faculty was not very much larger than the desired sample size for the study, all students from Year I to internship were enrolled into the study. Students were divided into two main categories, based on their current training level: (i) pre-clinical (Year I and Year II) and (ii) clinical (Year III, Year IV and internship).

A questionnaire was developed by reviewing relevant literature and previously used standardized instruments and protocols. It contained 100 questions, most of which were closed-ended with pre-coded responses. The questions were divided into four sections: (i) socio-demographic characteristics; (ii) alcohol and drug use; (iii) *khat *chewing habit; and (iv) smoking status. Substance use in this study is defined as the use of alcohol, *khat *and/or tobacco by medical students either in lifetime, in the past 12 months or in the past month/week. The survey questionnaire was constructed and administered in English since the medium of instruction in Ethiopia particularly in high schools and Universities is in English. The questionnaire was pre-tested in paramedical students, and appropriate revisions were made before being used for actual data collection.

Data were collected through self-administration of the questionnaires after gathering students in the lecture halls of the Faculty and explaining the purpose of the study. After the distribution of the questionnaire, the students were oriented on the questionnaire, the type of sections and the number of questions contained in it. Finally, instructions on how to properly fill the questionnaire, particularly how to follow skip patterns, were given to the students. The questionnaires were then collected back after they were completed by the end of the session; and checked and edited for completeness.

The data were entered into Epi Info version 6.04d and transferred to SPSS version 15 (SPSS Inc, Chicago, IL, USA) software program for analysis. Data entry was done by an experienced data clerk at the School of Public Health. Data cleaning, processing, preliminary analysis and final write-up were done by the research team. Descriptive statistics such as means for continuous and proportion for categorical variables including cross-tabulations were used for data summarization. Differences in proportions were compared for significance using Chi Square test, with significance level set at p < 0.05. When the assumptions of the Chi Square test were not fulfilled, we used the Fisher exact test.

Finally, multivariate logistic regression analyses were used to identify factors associated with substance use by controlling for the effect of potential confounding variables. Alcohol, *khat *and tobacco intake were included in the logistic model mainly as dependent variables, while the following factors were included in the model as independent variables: gender, age, religion, source of family income, rural-urban background, medical education status, parental and friends' substance use. Adjusted odds ratios (ORs) and their 95% confidence intervals were reported. The likelihood ratio statistic was used to test the evidence for interaction between the risk factors such as friends' and parental use of substances and the covariate gender.

This study was reviewed and approved by the Institutional Review Board of the Faculty of Medicine at Addis Ababa University. All responsible people in the Faculty were informed about this study. Participation of the students in this study was voluntary and verbal informed consent was obtained from each participant before data collection. Students were informed that questionnaires were anonymous and confidential. Names of the students were not recorded anywhere on the questionnaire and measures were taken to ensure the respect, dignity and freedom of each student participating in the study. Appropriate measures were also taken to ensure confidentiality of information both during and after data collection.

## Results

The final sample included 622 undergraduate medical students with complete data out of 632 students participated in the study. Ten filled questionnaires were discarded due to data incompleteness. The response rate was 78% (86% pre-clinical vs. 62% clinical students) from a total of 800 registered medical students from Year I to internship program at the Faculty by the time of the survey. The main reason for non-participation was absenteeism on the day of the data collection mainly due to various clinical practices at different hospitals in Addis Ababa.

Table 1 displays the socio-demographic characteristics of the sample by sex. The sample includes more men (68.5%) than women, which was consistent with the registrar's sex composition report of students. The overall age of the sample ranged from 18 to 39 years, with the majority (62%) being between 20 to 24 years. About 63% and 20% of the sample were Orthodox and Protestant Christians, respectively, followed by Muslim (14%). Only 2.1% of the respondents were either ever married or cohabited.

**Table 1:** Socio-demographic characteristics of the students by sex, June 2009

	Sex		
		
Characteristics	Male, n (%)	Female, n (%)	Total, n (%)
Age in years			
15-19	148 (34.7)	77 (39.3)	225 (36.2)
20-24	265 (62.2)	118 (60.2)	383 (61.6)
25 or more	13 (3.1)	1 (0.5)	14 (2.3)
Religion			
Orthodox Christian	264 (62.0)	125 (63.8)	389 (62.5)
Protestant Christian	83 (19.5)	41 (20.9)	124 (19.9)
Muslim	61 (14.3)	25 (12.8)	86 (13.8)
Other	18 (4.2)	5 (2.5)	23 (3.8)
Marital status			
Never married	415 (97.4)	194 (99.0)	609 (97.9)
Ever married/cohabiting	11 (2.6)	2(1.0)	13 (2.1)
Completed high school in Addis Ababa			
Yes	210 (49.3)	166 (84.7)	376 (60.5)
No	216 (50.7)	30 (15.3)	246 (39.5)
Original background			
Urban	311 (73.0)	187 (95.4)	498 (80.1)
Rural	115 (27.0)	9 (4.6)	124 (19.9)
Medical education status			
Year I	229 (53.8)	83 (42.3)	312 (50.2)
Year II	89 (20.9)	46 (23.5)	135 (21.7)
Year III	38 (8.9)	31 (15.8)	69 (11.1)
Year IV	47 (11.0)	34 (17.3)	81 (13.0)
Internship	23 (5.4)	2 (1.0)	25 (4.0)
Mother's educational level			
Illiterate	173 (40.6)	30 (15.3)	203 (32.6)
Elementary to grade 12	96 (22.5)	36 (18.4)	132 (21.2)
12^th ^grade complete plus diploma	113 (26.5)	83 (42.3)	196 (31.5)
First degree or above	44 (10.3)	47 (24.0)	91 (14.6)
Father's educational level			
Illiterate	138 (32.4)	18 (9.2)	156 (25.1)
Elementary to grade 12	92 (21.6)	36 (18.4)	128 (20.6)
12^th ^grade complete plus diploma	90 (21.1)	50 (25.5)	140 (22.5)
First degree or above	106 (24.9)	92 (46.9)	198 (31.8)
Family's main source of income			
Government employee	123 (28.9)	67 (34.2)	190 (30.5)
Business	97 (22.8)	65 (33.2)	162 (26.0)
Agriculture-based	126 (29.6)	8 (4.1)	134 (21.5)
NGO/private firm employee	37 (8.7)	40 (20.4)	77 (12.4)
Other	43 (10.1)	16 (8.2)	59 (9.5)

Total, N (%)	426 (68.5%)	196 (31.5%)	622 (100%)

With regard to the area of original residence, most students (80%) were from urban background and the remaining (20%) from rural areas (Table 1). About 95% of the females, compared with 73% of males, were from urban backgrounds. Similarly, about 61% of the students completed their high school education in Addis Ababa and the remaining (40%) finished their education in high schools all over around the country out of the capital. With regard to their medical training status, about 72% of them were pre-clinical (Year I and II), while 28% were clinical students (Year III and IV) including internship program. The number of medical students by the training years showed a recent massive enrollment of students into the medical school.

The parental education of the respondents revealed that 33% of the mothers and 25% of the fathers were reported to be illiterate, with only 15% of the mothers and 32% of the fathers with first degree or above (Table 1). Government employment (31%), business (26%), agriculture-based (22%), and NGO/private firm employee (12%) were the main reported source of parents' income. Students were also asked about their monthly pocket money. Although 23% of the students did not report their estimated monthly pocket money; of those who revealed it, 45% reported Birr 100-299 and 39% indicated Birr 300 or more. The average monthly income reported was Birr 246 (228 for males and 292 for females), and the overall median was Birr 100. The average monthly pocket money reported by female students was much higher than that reported by males.

Table 2 displays lifetime and past-year/month/week prevalence of alcohol use by sex. Lifetime use of alcohol was reported by about 31% (95% CI = 28-35) of the respondents and 22% reported drinking alcohol in the past year. There were significant differences between males and females with respect to alcohol use behavior; for example, about 35% of males reported lifetime alcohol consumption compared to 22% female students (p = 0.001). A greater number of males than females reported alcohol use over the past-year and past-month, showing a statistically significant difference (p = 0.002 and p = 0.014, respectively). About 9% of students reported drinking alcohol in the past month. A notable difference, though not statistically significant, was also observed between males (6.3%) and females (3.1%) with regard to the past-week alcohol use (p = 0.090). Furthermore, about 6% of male and 2% of female students reported that they were current users of alcohol (p = 0.045). In general, men were more likely to consume alcohol than females. The types of alcohol most frequently consumed by male students were beer, draft, wine and *tela/areqe/teji*, while wine, whisky and beer were the three most commonly consumed alcohols by female students.

**Table 2:** Lifetime and current use of alcohol, and the most commonly consumed types by sex, June 2009

	Sex			
			
Alcohol use	Male, n (%)	Female, n (%)	Total, n (%)	p-value
Ever drunk alcohol				
Yes	151 (35.4)	44 (22.4)	195 (31.4)	**0.001**
No	275 (64.6)	152 (77.6)	427 (68.6)	
Drunk alcohol within the last 12 months				
Yes	107 (25.1)	28 (14.3)	135 (21.6)	**0.002**
No	319 (74.9)	168 (85.7)	487 (78.4)	
Drunk alcohol within the last 30 days				
Yes	48 (11.3)	10 (5.1)	58 (9.3)	**0.014**
No	378 (88.7)	186 (94.6)	564 (90.7)	
Consumed alcohol within the last 7 days				
Yes	27 (6.3)	6 (3.1)	33 (5.3)	0.090
No	399 (93.7)	190 (96.9)	589 (94.7)	
Currently drinking alcohol				
Yes	24 (5.6)	4 (2.0)	28 (4.5)	**0.045**
No	402 (94.4)	192 (98.0)	594 (95.5)	

Total, N (%)	426 (68.5)	196 (31.5)	622 (100)	

Socio-demographic and behavioral correlates assumed to be associated with alcohol use among the study participants were assessed using logistic regression (Table 3). Compared to female, being male was strongly associated with alcohol use within the last 12 months (adjusted OR = 2.14, 95% CI = 1.22-3.76). Orthodox Christianity was also strongly associated with alcohol use in the past year (adjusted OR = 4.67, 95% CI = 2.05-10.64). The odds of alcohol consumption in the past year among Muslim students were significantly lower compared to students of other religions. Those students whose fathers currently drinks alcohol were more likely to use alcohol in the past year as compared to those students whose fathers did not drink alcohol (adjusted OR = 2.60, 95% CI = 1.50-4.51). The odds of alcohol use over the past year were 2.47 fold higher among students whose friends currently consume alcohol compared to students whose friends did not (adjusted OR = 2.47, 95% CI = 1.50-4.08).

**Table 3:** Socio-demographic and behavioral correlates of alcohol use within the last 12 months among undergraduate medical students, June 2009

	Alcohol use within the last 12 months		
		
Factors	No, n (%)	Yes, n (%)	Adjusted* OR (95% CI)
Sex			
Female	168 (85.7)	28 (14.3)	1.00 (Reference)
Male	319 (74.9)	107 (25.1)	**2.14 (1.22, 3.76)**
Age in years			
15-19	178 (79.1)	47 (20.9)	1.00 (Reference)
20 or more	309 (77.8)	88 (22.2)	1.07 (0.58, 1.98)
Religion			
Other Christian	121 (91.7)	11 (8.3)	1.00 (Reference)
Orthodox Christian	278 (71.5)	111 (28.5)	**4.67 (2.05, 10.64)**
Muslim	80 (93.0)	6 (7.0)	**0.20 (0.04, 0.92)**
Other	8 (53.3)	7 (46.7)	**13.64 (2.58, 72.10)**
Main source of family's income			
Government employee	142 (74.7)	48 (25.3)	1.00 (Reference)
Business	116 (71.6)	46 (28.4)	1.78 (0.96, 3.31)
Agriculture-based	121 (90.3)	13 (9.7)	0.34 (0.10, 1.12)
Other	108 (79.4)	28 (20.6)	1.12 (0.59, 2.16)
Original background			
Rural	106 (85.5)	18 (14.5)	1.00 (Reference)
Urban	381 (76.5)	117 (23.5)	0.94 (0.32, 2.76)
Medical education status			
Pre-clinical	363 (81.2)	84 (18.8)	1.00 (Reference)
Clinical	124 (70.9)	51 (29.1)	0.98 (0.51, 1.90)
Father currently drinks alcohol			
No	381 (83.7)	74 (16.3)	1.00 (Reference)
Yes	106 (63.5)	61 (36.5)	**2.60 (1.51, 4.51)**
Mother currently drinks alcohol			
No	442 (79.6)	113 (20.4)	1.00 (Reference)
Yes	45 (67.2)	22 (32.8)	0.82 (0.40, 1.72)
Friend currently drinks alcohol			
No	340 (88.1)	46 (11.9)	1.00 (Reference)
Yes	147 (62.3)	89 (37.7)	**2.47 (1.50, 4.08)**
Used *khat *within the last 12 months			
No	477 (82.4)	102 (17.6)	1.00 (Reference)
Yes	10 (23.3)	33 (76.7)	**15.11 (4.24, 53.91)**
Ever smoked cigarette			
No	474 (83.5)	94 (16.6)	1.00 (Reference)
Yes	13 (24.1)	41 (75.9)	**8.65 (3.48, 21.50)**

Total, N (%)	487 (78.3)	135 (21.1)	

*Adjusted for sex, age, religion (4 levels), income (4 levels), background, education, alcohol intake of friends and parents, *khat *and cigarette intake.

*Khat *use within the past 12 months was strongly and positively associated with alcohol consumption in the past year (adjusted OR = 15.11, 95% CI = 4.24-53.91) (Table 3). About 77% of respondents reporting alcohol use in the past year had chewed *khat*, as opposed to only 23.3% of those who had not consumed alcohol. Similarly, ever use of cigarette was also significantly associated with alcohol consumption (adjusted OR = 8.65, 95% CI = 3.48-21.50), 76% of those who consumed alcohol in the past year ever used cigarette compared to 24% of those who had not ever used cigarette. The likelihood ratio test suggests an evidence of interaction between friends' use of alcohol and gender (p = 0.026) and between parental use of alcohol and gender (p = 0.003). That means the estimated effect of friends' and parental use of alcohol on the participants use of alcohol varies by gender.

About 14% (95% CI = 11.50-17.10) of the participants reported lifetime use of *khat *(18% males vs. 6% females, p < 0.001) (Table 4). Only 7% of the participants reported the use of *khat *within the last 12 months and about 4% did it in the past week. Only about 2% of the total respondents reported the current use of *khat*. Of the total respondents, the proportion of students who had ever smoked and the proportion who reported current cigarette smoking by the time of the study was 9% and 1.8%, respectively. It was noted that the use of *khat *and cigarette smoking was reported to be higher among male than female students. The main reasons reported for chewing *khat *included for effective reading and studying (68%), for enjoyment (63%) and to get rid of sleeplessness (43%). The students also reported that alcohol consumption (75%) and cigarette smoking (63%) were the main additional substances used after *khat *chewing.

**Table 4:** Respondent's lifetime and current use of *khat *and cigarette smoking by sex, June 2009

	Sex			
			
*Khat *use and cigarette smoking habit	Male, n (%)	Female, n (%)	Total, n (%)	p-value
Ever chewed *khat*				
Yes	77 (18.1)	11 (5.6)	88 (14.1)	
No	349 (81.9)	185 (94.4)	534 (85.9)	**< 0.001**
Chewed *khat *within the last 12 months				
Yes	40 (9.4)	3 (1.5)	43 (6.9)	
No	386 (90.6)	193 (98.5)	579 (93.1)	**< 0.001**
Chewed *khat *within the last 30 days				
Yes	21 (4.9)	2 (1.0)	23 (3.7)	
No	405 (95.1)	194 (99.0)	599 (96.3)	**0.016**
Chewed *khat *within the last 7 days				
Yes	15 (3.5)	2 (1.0)	17 (2.7)	
No	411 (96.5)	194 (99.0)	605 (97.3)	0.075
Currently chewing *khat*				
Yes	12 (2.8)	2 (1.0)	14 (2.3)	
No	414 (97.2)	194 (99.0)	608 (97.7)	0.244
Ever smoked cigarette				
Yes	45 (10.6)	9 (4.6)	54 (8.7)	
No	381 (89.4)	187 (95.4)	568 (91.3)	**0.014**
Currently smoking cigarette				
Yes	10 (2.3)	1 (0.5)	11 (1.8)	
No	416 (97.7)	195 (99.5)	611 (98.2)	0.187

**Total, n (%)**	426 (68.5)	196 (31.5)	622	

Table 5 shows the socio-demographic and behavioral correlates assumed to be associated with *khat *use among medical students in the past 12 months. Results from a multiple logistic regression analysis indicated that being Muslim was strongly and positively associated with *khat *use in the past year (adjusted OR = 8.18, 95% CI = 1.55-43.25), but students who reported business as their parent's main source of income were less likely to consume *khat *than students who reported other source of income for their parents (adjusted OR = 0.27, 95% CI = 0.08-0.86). Students who reported having a friend who currently chew *khat *were about eight times more likely to chew *khat *than those students who reported no such friend (adjusted OR = 8.08, 95% CI = 2.84-22.98). Likewise, a student who consumed alcohol in the past 12 months had the odds of 17-fold increase to use *khat *in the past year (adjusted OR = 16.99, 95% CI = 4.61-62.64). Similarly, ever use of cigarette was strongly associated with *khat *consumption (adjusted OR = 7.63, 95% CI = 2.81-20.71).

**Table 5:** Socio-demographic and behavioral correlates of *khat *use within the last 12 months among undergraduate medical students, June 2009

Factors	*Khat *use within the last 12 months		Adjusted* OR (95% CI)
		
	No, n (%)	Yes, n (%)	
Sex			
Female	193 (98.5)	3 (1.5)	1.00 (Reference)
Male	386 (90.6)	40 (9.4)	3.28 (0.80, 13.54)
Age in years			
15-19	213 (94.7)	12 (5.3)	1.00 (Reference)
20 or more	366 (92.2)	31 (7.8)	0.64 (0.18, 2.30)
Religion			
Other Christian	127 (96.2)	5 (3.8)	1.00 (Reference)
Orthodox Christian	366 (94.1)	23 (5.9)	0.62 (0.15, 2.61)
Muslim	74 (86.1)	12 (14.0)	**8.18 (1.55, 43.25)**
Other	12 (80.0)	3 (20.00)	1.42 (0.13, 13.82)
Main source of family's income			
Government employee	172 (90.5)	18 (9.5)	1.00 (Reference)
Business	149 (92.0)	13 (8.0)	**0.27 (0.08, 0.86)**
Agriculture-based	128 (95.5)	6 (4.5)	0.77 (0.09, 6.50)
Other	130 (95.6)	6 (4.4)	0.51 (0.14, 1.87)
Medical education status			
Pre-clinical	426 (95.3)	21 (4.7)	1.00 (Reference)
Clinical	153 (87.4)	22 (12.6)	2.04 (0.61, 6.78)
Original background			
Rural	116 (93.6)	8 (6.5)	1.00 (Reference)
Urban	463 (93.0)	35 (7.0)	0.77 (0.11, 5.29)
Friend currently uses *khat*			
No	436 (96.5)	16 (3.5)	1.00 (Reference)
Yes	143 (84.1)	27 (15.9)	**8.08 (2.84, 22.98)**
Father currently chews khat			
No	534 (93.2)	39 (6.8)	1.00 (Reference)
Yes	45 (91.8)	4 (8.2)	1.75 (0.44, 7.03)
Mother currently chews *khat*			
No	560 (93.1)	42 (6.9)	1.00 (Reference)
Yes	19 (95.0)	1 (5.0)	1.32 (0.12, 14.74)
Consumed alcohol within the last 12 months			
No	477 (97.9)	10 (2.1)	1.00 (Reference)
Yes	102 (75.6)	33 (24.4)	**16.99 (4.61, 62.64)**
Ever smoked cigarette			
No	551 (97.0)	17 (3.0)	1.00 (Reference)
yes	28 (51.9)	26 (48.2)	**7.63 (2.81, 20.71)**

Total, N (%)	579 (93.1)	43 (6.9)	

*Adjusted for sex, age, religion (4 levels), income (4 levels), education, background, *khat *intake of friends and parents, alcohol and cigarette intake.

The multiple logistic regression model also indicates that various socio-demographic characteristics and other behavioral aspects are associated with lifetime smoking status of medical students (Table 6). Students whose parent's income was based on agriculture reported the odds of 0.15-fold decrease in lifetime use of cigarette (adjusted OR = 0.12, 95% CI = 0.01-1.02) compared to others, but it was not statistically significant. Similarly, students with smoker friends were about four times more likely to ever smoke as compared to those whose friends are non-smokers (adjusted OR = 3.89, 95% CI = 1.83-8.30). Likewise, study participants who reported the use of alcohol and *khat *in the last 12 months reported high odds of smoking cigarettes, adjusted OR = 8.56, 95% CI = 3.44-21.35 and adjusted OR = 8.28, 95% CI = 3.24-21.13, respectively. Students whose fathers were reported to be smokers were more likely to smoke, but it was not statistically significant. Sex, age, religion, background and training status of the students were not significantly associated with lifetime smoking. No evidence of interaction was observed between the covariate gender and the main risk factors of friends' use of tobacco and alcohol use.

**Table 6:** Socio-demographic and behavioral correlates of cigarette smoking among undergraduate medical students, June 2009

	Ever smoked		
		
Factors	No, n (%)	Yes, n (%)	Adjusted* OR (95% CI)
Sex			
Female	187 (95.4)	9 (4.6)	1.00 (Reference)
Male	381 (89.4)	45 (10.6)	1.45 (0.57, 3.67)
Age in years			
15-19	209 (92.9)	16 (7.1)	1.00 (Reference)
20 or more	359 (90.4)	38 (9.6)	1.00 (0.35, 2.64)
Religion			
Other Christian	124 (93.9)	8 (6.1)	1.00 (Reference)
Orthodox Christian	356 (91.5)	33 (8.5)	0.47 (0.15, 1.45)
Muslim	77 (89.3)	9 (10.5)	0.90 (0.23, 3.53)
Other	11 (73.3)	4 (26.7)	3.04 (0.34, 26.94)
Main source of family's income			
Government employee	169 (88.9)	21 (11.1)	1.00 (Reference)
Business	140 (86.4)	22 (13.6)	1.15 (0.46, 2.89)
Agriculture-based	131 (97.8)	3 (2.2)	0.12 (0.01, 1.02)
Other	128 (94.1)	8 (5.9)	0.61 (0.21, 1.80)
Original background			
Rural	118 (95.2)	6 (4.8)	1.00 (Reference)
Urban	450 (90.4)	48 (9.6)	0.69 (0.12, 4.10)
Medical education status			
Pre-clinical	419 (93.7)	28 (6.3)	1.00 (Reference)
Clinical	149 (85.1)	26 (14.9)	1.26 (0.46, 3.45)
Friend currently smoking			
No	434 (96.0)	18 (4.0)	1.00 (Reference)
Yes	18 (4.0)	434 (96.0)	**3.89 (1.83, 8.30)**
Father currently smoking			
No	544 (91.6)	50 (8.4)	1.00 (Reference)
Yes	24 (85.7)	4 (14.3)	2.57 (0.63, 10.56)
Consumed alcohol within the last 12 months			
No	474 (97.3)	13 (2.7)	1.00 (Reference)
Yes	94 (69.6)	41 (30.4)	**8.68 (3.52, 21.45)**
Used *khat *within the last 12 months			
No	551 (95.2)	28 (4.8)	1.00 (Reference)
Yes	17 (39.5)	26 (60.5)	**7.88 (3.11, 19.95)**

Total, N (%)	568 (91.3)	54 (8.7)	

*Adjusted for sex, age, religion (4 levels), income (4 levels), background, education, smoking status of friends and fathers, alcohol and *khat *intake.

## Discussion

This study clearly indicates that substance use is becoming a concern among undergraduate medical students. About 22% of the participants reported alcohol drinking and 7% used *khat *in the past 12 months; while lifetime cigarette smoking was reported by 9% of the students. The prevalence occurs across all years of study, although it is higher among clinical students than pre-clinical ones.

Our reported rate of alcohol consumption among medical students was lower than that reported by other studies in a similar study population. A study in Ethiopia based on a large sample of youth aged between 15 and 24 years showed that about 9% of in-school youths consumed alcohol either on a weekly or daily basis in Ethiopia [14]. In the USA, a study among a cohort of medical students found that 78% of them reported drinking alcohol in the past month and 34% engaged in excessive drinking [24]. A study in Sweden showed that more than 90% of university students consumed alcohol during the preceding three months and 39% of males and 20% of females involved in heavy episodic drinking at least once a week [25].

Studies on *khat *use among college students in general and medical students in particular are very scanty. Our findings indicate that *khat *use in the past 12 months and one month was 7% and 4%, respectively, significantly lower than the 18% current *khat *chewing report from four college students in northwest Ethiopia [13]. A study that assessed the association of *khat*/alcohol use and risky sexual behavior among in-school and out-of-school youth in Ethiopia found that about 9% of in-school youth reported current intake of *khat *either weekly or every day [14]. The same study revealed a significant and positive association between *khat*/alcohol use and unprotected sex, leading to exacerbation of HIV transmission.

There have been several studies on cigarette smoking among young adults in developed countries, but such studies are scantly in developing countries. In northwest Ethiopia, lifetime and current use of cigarette smoking was reported by 13% and 8% of college students, respectively [13]. In a study of in-school rural Zambian adolescents, the prevalence of lifetime cigarette smoking was reported to be 27% [26], much higher than our findings. Another study conducted among 18-23 year-old college students in India found 14% prevalence of cigarette smoking [27].

Results from this study provide important insights into the magnitude of substance use and its associated risk factors. The present study found that peer's influence was one of the important factors to influence the students to practice substance use. We found that those students who reported friend's use of alcohol, *khat *and cigarette smoking were more likely to be users of either one or all of the above substances than those students whose friends were non-users. Previous studies also identified that friends' and parental use of substances were strongly associated with the use of substances among adolescents, indicating the influence of peer pressure [26,28,29]. Our findings also indicate that students who reported alcohol use of their fathers were more likely to consume alcohol than their counterparts. Similar findings from other studies also showed that adolescents whose parents smoked were more likely to have smoked [26,30].

Our findings show that being a Muslim was strongly and positively associated with *khat *use, but inversely related with alcohol use in the last 12 months. In contrast, belonging to Orthodox Christianity was strongly and positively associated with alcohol use. In São Paulo, a study indicated that practicing a religion among university students was found as a protective factor of psychoactive substance use [31]. The same study also found that monthly family income was significantly associated with alcohol and drug use. However, the present study reported that family's source of income was not as important as the influence by other factors. In the present study, sex was related with alcohol use and cigarette smoking, yet it was not related with *khat *use.

The present findings show that students who consume alcohol or use *khat *are at an increased risk of cigarette smoking. Consistent to our findings, several studies documented an association between cigarette smoking and substance use among adolescents. In Bolivia, cigarette smoking was consistently higher among those who consumed alcohol or other drugs at least once, 24% of those reporting marijuana use also reported having used tobacco during the preceding 30 days as opposed to only 2% of those who had not used marijuana [32]. In Pakistan, students whose friends are smokers were more likely to smoke than those whose friends are non-smokers [28].

This study is not free of limitations. First, the study used a descriptive single cross-sectional design that can not establish trends and causality between substance use and potential risk factors. Second, the males are over represented in this sample largely because of disproportionate enrollment of both sexes into the medical school. Small sample sizes, particularly among females and substance users, contributed to wider confidence intervals with low precision. Our sample may also not be representative of the population since only one medical school was purposefully included in the study. Third, the data was collected based on self-report of the students and may be subjected to recall bias and under-reporting of substance use due to social desirability bias. Finally, the lifetime report of cigarette smoking in the current study is not the most sensitive indicator. Despite the limitations, these findings indicate a need to educate medical students regarding substance use and its consequences.

## Conclusion

This study has revealed that the magnitude of substance use among undergraduate medical students was considerable, although not very high, but lower than the findings of other studies that reported for adolescents and young adults. This study also indicated that substance use is significantly associated with friends or parental use of substances. Alcohol consumption or *khat *use has been significantly associated with cigarette smoking. Understanding factors associated with substance use is the first step for designing and implementing comprehensive anti-substance use interventions that simultaneously prevent multiple risk factors among medical students. The medical profession in turn has an ethical obligation to ensure that its practitioners can discharge their duties and responsibilities for the welfare and safety of the population.

## Recommendations

As our study focuses on undergraduate medical students, the findings would be helpful to initiate effective substance use control programs in medical schools and would also serve as a stimulator to conduct further studies on this topic in Ethiopia.

## Competing interests

The authors declare that they have no competing interests.

## Authors' contributions

WD involved in proposal writing, designed the study and participated in all implementation stages of the project. He analyzed the data, drafted and finalized the write up of the paper. AA conceived the original idea, involved in proposal writing, got funding for the study and participated in all stages of the project implementation and write up of the paper. All authors read and approved the final manuscript.

## Pre-publication history

The pre-publication history for this paper can be accessed here:

http://www.biomedcentral.com/1471-2458/11/660/prepub
